# Comparative genome-wide transcriptome analysis of *Vitis vinifera* responses to adapted and non-adapted strains of two-spotted spider mite, *Tetranyhus urticae*

**DOI:** 10.1186/s12864-016-2401-3

**Published:** 2016-01-22

**Authors:** Jose Díaz-Riquelme, Vladimir Zhurov, Cristina Rioja, Ignacio Pérez-Moreno, Rafael Torres-Pérez, Jérôme Grimplet, Pablo Carbonell-Bejerano, Sabina Bajda, Thomas Van Leeuwen, José Miguel Martínez-Zapater, Miodrag Grbic, Vojislava Grbic

**Affiliations:** Department of Biology, The University of Western Ontario, 1151 Richmond Street, London, ON N6A5B7 Canada; Instituto de Ciencias de la Vid y del Vino, 26006 Logroño, Spain; University of La Rioja, 26006 Logroño, Spain; Department of Crop Protection, Ghent University, B-9000 Ghent, Belgium; Institute for Biodiversity and Ecosystem Dynamics, University of Amsterdam, 1098 XH Amsterdam, The Netherlands

**Keywords:** *Vitis vinifera*, *Tetranychus urticae*, Constitutive defense responses, Induced defense responses, Adaptation, Pest, Herbivory

## Abstract

**Background:**

The two-spotted spider mite, *Tetranychus urticae,* is an extreme generalist plant pest. Even though mites can feed on many plant species, local mite populations form host races that do not perform equally well on all potential hosts. An acquisition of the ability to evade plant defenses is fundamental for mite’s ability to use a particular plant as a host. Thus, understanding the interactions between the plant and mites with different host adaptation status allows the identification of functional plant defenses and ways mites can evolve to avoid them.

**Results:**

The grapevine genome-wide transcriptional responses to spider mite strains that are non-adapted and adapted to grapevine as a host were examined. Comparative transcriptome analysis of grapevine responses to these mite strains identified the existence of weak responses induced by the feeding of the non-adapted strain. In contrast, strong but ineffective induced defenses were triggered upon feeding of the adapted strain. A comparative meta-analysis of *Arabidopsis*, tomato and grapevine responses to mite feeding identified a core of 36 highly conserved genes involved in the perception, regulation and metabolism that were commonly induced in all three species by mite herbivory.

**Conclusions:**

This study describes the genome-wide grapevine transcriptional responses to herbivory of mite strains that differ in their ability to use grapevine as a host. It raises hypotheses whose testing will lead to our understanding of grapevine defenses and mite adaptations to them.

**Electronic supplementary material:**

The online version of this article (doi:10.1186/s12864-016-2401-3) contains supplementary material, which is available to authorized users.

## Background

Plants have evolved both constitutive and induced defenses to deter herbivory. Constitutive defenses include various physical and chemical barriers that exist even in the absence of herbivore challenge, while induced defenses occur upon herbivore attack and result in the biosynthesis of defense compounds (metabolites and defense proteins) that reduce the performance of the herbivore through toxicity, anti-feeding effects and attraction of natural predators. Induced defenses also lead to changes in physical properties of plant tissues, of particular importance in defenses against pathogens [1].

The two-spotted spider mite (TSSM), *Tetranychus urticae*, is a polyphagous pest that feeds on more than 1,100 plant species [2, 3]. The ability of TSSM to feed on such a wide range of plant species implies that it is capable of evading diverse plant defenses. So far, we have characterized responses of *Arabidopsis* and tomato plants to TSSM feeding [4, 5]. In both cases, TSSM induced a conserved set of genes associated with the biosynthesis of plant hormone jasmonic acid (JA) and its signaling, but a highly divergent set of JA-regulated defense genes. For example, in *Arabidopsis*, indole glucosinolates were the most prominent defense compounds, while in tomato TSSM induced the expression of genes encoding enzymes predicted to interfere with mite’s ability to effectively digest plant nutrients [4, 5]. The ability of TSSM to avoid diverse plant defense compounds is associated with the expansion of gene families encoding detoxification enzymes and transporters, as well as acquisition of genes from various (micro)organisms through the horizontal gene transfers [6–8]. Even though TSSMs can feed on a wide array of plants, individual TSSM populations do not perform equally well on all potential hosts. Intraspecific variation leading to locally adapted populations has been demonstrated to be one of the mechanisms underlying the evolution of TSSM’s host range [9–12]. Conceptually, spider mite adaptation to a new host can be based on mite’s ability to evade physical/anatomical barriers to its feeding, or in the case of defenses that rely on plant defense compounds, to detoxify them or to attenuate their synthesis. Even though mite adaptation to a particular plant host is important for the understanding of its polyphagous nature, mechanisms of adaptation and molecular patterns of herbivore and/or host associated with mite adaptation have been described in only few instances. For example, *T. lintearius,* a specialist on gorse (*Ulex europaeus)* evades host constitutive defenses by adapting its feeding behavior. Gorse has a thick cuticle that hinders mite stylet penetration, however, *T. linearius* bypasses this barrier by inserting its stylet into the leaf mesophyll through the stomatal aperture [13]. In addition, it has been shown that TSSMs can adapt to beans that constitutively synthesize cyanogenic glucosides by acquiring an ability to detoxify them through the overexpression of a member of the cysteine synthase family [8]. Furthermore, plant and mite responses indicating involvement of both detoxification and attenuation of induced plant defenses have been described for mite adaptation to cultivated tomato, a plant host that relies on induced defenses to deter mite herbivory [4, 10, 12, 14]. The extent of chemical versus physical constitutive defenses across mite potential hosts is not known, nor is it clear if detoxification, rather than attenuation of metabolite biosynthesis, is a prevailing pattern of mite adaptation to hosts that rely on induced accumulation of defense metabolites to deter herbivory, and if the reverse is true in cases of mite adaptations to host proteaceous defense compounds (e.g., protease inhibitors). Therefore, description of multitude of plant interactions with adapted and non-adapted mite strains is required to gain insights into functional plant defenses and the ways mites can evolve to evade them.

Grapevine is a model plant for fruit-bearing perennial crops with established genomic resources [15]. Several genome-wide studies of transcriptional changes over developmental and fruit maturation stages, as well as responses to abiotic stresses and fungal pathogens have been reported recently [16–21]. Grapevine responses to the specialist gall-forming aphid-like parasite phylloxera, *Daktulosphaira vitifoliae*, have been recently described [22], however, studies of grapevine responses to generalist herbivores are so far lacking. Here, we describe genome-wide grapevine transcriptional responses to herbivory of adapted and non-adapted spider mite strains in order to begin an understanding of the mechanism of TSSM adaptation to grapevine.

## Results

### Dispersal behavior and leaf damage analysis of London and Murcia TSSM strains on grapevine

In this study we used two strains of TSSM: a) the reference London strain that was originally collected from apple trees; it has been propagated on bean plants for the last ten years [6]; and b) the Murcia strain that was collected from a heavily infested vineyard in the region of Murcia, Spain; this strain was subsequently maintained on grapevine plants for more than two years. We developed and used inbred lines of both strains to minimize genetic variability of field-collected Murcia strain. In addition, to eliminate the physiological effects of plant hosts (bean versus grapevine), both strains were reared for two generations on a common host prior to their experimental application. As London strain was not able to survive on grapevine, we used beans to rear London and Murcia mites for two generations. To establish the relationship between these mite strains and grapevine as a host, we determined their dispersal patterns and leaf damage they caused upon feeding.

**Fig. 1:**
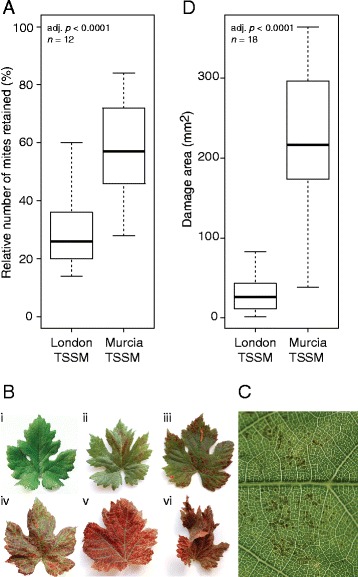
Performance of non-adapted London and adapted Murcia spider mite strains on grapevine. **a** Dispersal behavior of spider mites, as assessed by the total number of mites retained on the infested leaf after 24 h. **b** Progressive symptoms of feeding of the adapted mite on grapevine leaves (i-vi). **c** Close up of the grapevine leaf exposed to spider mite feeding for 24 h showing brown spots. **d** Extent of damage area of brown spots of grapevine leaves exposed to 50 mites for 24 h

Mite behavior is affected by plant host, such that if a host is favorable, TSSMs have a tendency to remain within the infestation area. However, if the host is unfavorable, TSSMs tend to disperse and in the extreme cases to leave the plant [23, 24]. Grapevine is considered a non-favourable host for spider mites [25]. Consistently, only 28 % of London mites were retained on the leaf they were initially placed on, while Murcia strain had significantly greater preference to grapevine with 58 % retention after 24 h, Fig. 1a. Since differential retention of TSSM on leaves is expected to impact the grapevine responses to mite herbivory, we used a combination of wet cotton and lanolin to create a barrier at the leaf petiole in order to confine mites to inoculated leaves in all subsequent experiments (see Additional file 1).

The most common symptom of TSSM feeding is the formation of chlorotic spots on the host plant leaves [4, 5]. However, mite herbivory on grapevine does not result in the formation of macroscopic chlorotic spots. Rather, mite feeding induces an accumulation of red/brown spots that is referred to as leaf bronzing [26]. Short-term symptoms of mite feeding (24 h) can be seen as individual brown spots, but longer-term, leaves turn red, desiccate and finally abscise, Fig. 1b, c. Thus, to measure the intensity of mite feeding we determined the area of brown spots that formed on leaves infested with 50 female mites of London or Murcia strains upon 24 h of herbivory. Leaves inoculated with Murcia mites developed significantly greater area of brown spots relative to London mites (220 vs. 29 mm^2^ respectively, Fig. 1d). Individual spots induced by London or Murcia mites were similar in appearance (data not shown), indicating that Murcia mites feed more than London strain. Ability of the Murcia mite strain to successfully develop on grapevine, to display significantly higher retention and to feed intensively even when maintained on beans for two generation, indicates that this strain has intrinsic ability to reduce/eliminate restrictions imposed by the grapevine as a host. We consider this strain adapted to grapevine. In contrast, London strain is non-adapted to grapevine, as it cannot establish its population, tends to disperse and has limited feeding on grapevine.

### Induced grapevine responses to feeding of London and Murcia spider mite strains

**Fig. 2:**
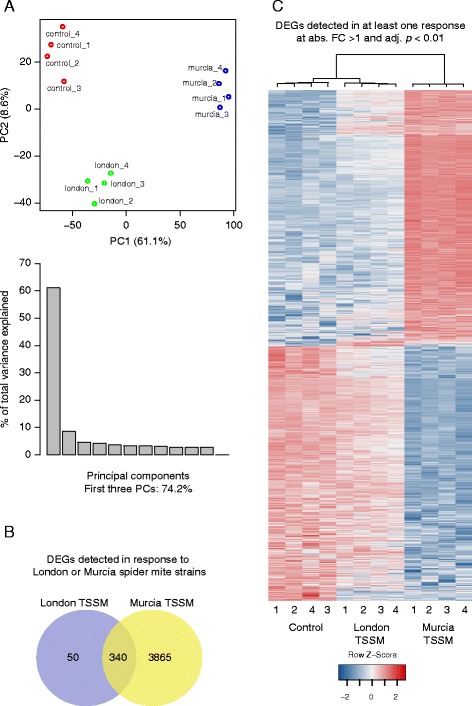
Grapevine transcriptional responses to feeding of non-adapted London and adapted Murcia spider mite strains. **a** Principal component analysis of expression measures data for non-adapted London and adapted Murcia TSSM strains. **b** Comparison of DEGs detected in response to London or Murcia TSSM strains. **c** Hierarchical clustering analysis of log_2_ Fold Changes exhibited by DEGs with absolute FC > 2 and BH-adjusted *P* < 0.01 detected upon feeding of non-adapted London and adapted Murcia TSSM strains for 24 h. The distance metric was Pearson’s *r*, and the hierarchical clustering method was average

Dispersal and damage analysis assays indicated that grapevine plants are efficient in deterring feeding of non-adapted London mites, but that their defenses are less effective against the adapted Murcia strain. To compare genome-wide transcriptional grapevine responses to feeding of London and Murcia mite strains, an RNA-Seq experiment was performed with *Vitis vinifera* cv. Tempranillo plantlets infested with either London or Murcia mite strains, and their transcriptional responses were measured 24 h later. Principal component analysis (PCA) identified robust effects of mite treatments corresponding to the first principal component that explained 61.1 % of total variance in the data, Fig. 2a. With an absolute fold change (FC) above 2 and Benjamini-Hochberg (BH) corrected *p* values below 0.01 we detected 390 differentially expressed genes (DEGs) in response to herbivory of the London strain and 4,205 DEG in response to the Murcia strain, Fig. 2b. In total, 4,255 DEG were detected in at least one response with about an equal proportion of up- and down-regulated genes, Additional file 2. Clustering analysis of voom-transformed DEG read counts demonstrated that grapevine responses to non-adapted London mites were minor and clustered closer to non-treated control samples than to the responses triggered by the adapted Murcia mite strain, Fig. 2c.

**Fig. 3:**
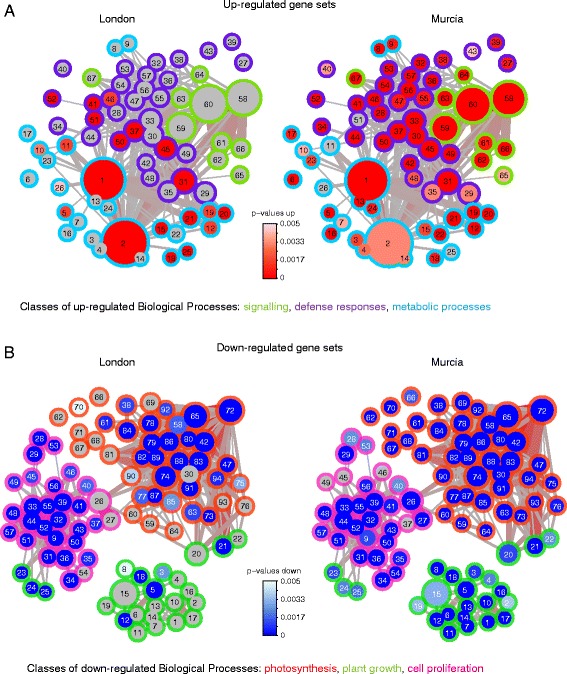
Gene set enrichment analysis of biological processes for differentially expressed genes (DEG) detected in grapevine responses to feeding of non-adapted London and adapted Murcia spider mite strains. Parametric analysis of gene set enrichment (PAGE) network based on Biological Processes (BP) Gene Ontology (GO) annotation with significantly enriched (**a**) up- and (**b**) down-regulated gene sets. Nodes represent gene sets, edges indicate the overlap in genes belonging to connected gene sets. Gene sets: blue – down-regulated, red – up-regulated, gray – not detected as differentially regulated. Size corresponds to number of genes in a given gene set (up-regulated gene sets – 15 to 568, down-regulated – 15 to 112), correspondence between node labels and GO Term/ID is provided in Data S3. The color (gray to red) and width of the edges correspond to an overlap size (up-regulated gene sets – 8 to 351, down-regulated – 8 to 55)

Gene Ontology (GO) and Gene Set Analysis (GSA) corroborated functional differences in grapevine responses induced by the London and Murcia mite strains. A union PAGE network of enriched biological processes consisted of 161 gene sets (67 up- and 94 down-regulated) represented by at least 15 DEGs, Fig. 3 (correspondence between node labels and GO Term/ID is provided in Additional file 3). Feeding of grapevine-adapted Murcia mites triggered up-regulation of a wide range of processes that can be broadly grouped in three classes: signaling (marked in green in Fig. 3a), defense responses (including jasmonic acid (JA), ethylene (ET), salicylic acid (SA) and abscisic acid (ABA) biosynthesis, signaling and responses, marked in violet in Fig. 3a) and metabolic processes (including amino acid metabolism and production of secondary metabolites and their transport; marked in cyan in Fig. 3a). In contrast, only a few gene sets were significantly up-regulated by the application of non-adapted London mites. None of the signaling processes were enriched, and only some defense responses (i.e. JA and ET biosynthesis (nodes 51, 52); responses to JA, ET and ABA (nodes 50, 46, 45 respectively); responses to wounding (node 37) and fungus (41), Additional file 3) and metabolic processes (oxidation-reduction processes, and the biosynthesis of phenylpropanoid metabolites coumarin and stilbene (nodes 19, 20), Additional file 3) were enriched. Down-regulated biological processes were associated with photosynthesis, plant growth and cell proliferation (outlined in red, green and magenta, respectively in Fig. 3b), all robustly affected by the feeding of the Murcia adapted mites. The application of London mites resulted in down-regulation of photosynthesis and cell proliferation; however, the plant growth gene sets were mostly not significantly affected. Thus, the ability of grapevine to deter feeding of the London non-adapted mite strain was associated with weak induced responses, while feeding of Murcia mites, adapted to grapevine as a host, induced robust responses.

### Grapevine responses to feeding of non-adapted London mite strain

London mites induced marginal grapevine responses and clustered with untreated control state. However, the PCA analysis identified these responses as distinct. Thus, we further analyzed grapevine responses to non-adapted mites in search for induced responses that could explain plant resistance. We first looked at a set of 50 genes that were differentially expressed only upon feeding of London non-adapted mites, Fig. 2 and Additional file 4. Eighteen of these genes were down-regulated, including genes homologous to *Arabidopsis NINE-CIS-EPOXYCAROTENOID DIOXYGENASE 3 (NCED3)* and *ABA INSENSITIVE 1 (ABI1)* that are involved in ABA biosynthesis and signal transduction, as well as two genes encoding disease resistance proteins that may be involved in innate immune responses. Of thirty-two up-regulated genes, two are associated with thiamine biosynthesis process and additional two with UDP-glucose transport. Several other transcripts encoding signaling proteins (Ca^2+^ binding, tetratricopeptide repeat (TPR)-containing) were also differentially expressed. Some of these transcripts were also detected in response to abiotic stresses [27]. However, differential expression of this set of 50 genes did not affect global changes in the expression of defense-related genes, thus, they unlikely impacted the grapevine defenses against the herbivory of the non-adapted London mites.

**Fig. 4:**
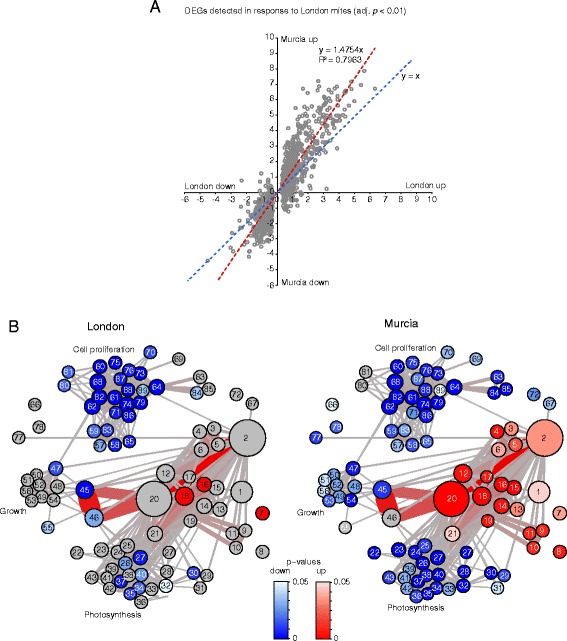
Analysis of differentially expressed genes detected in response to London spider mite strain with BH-adjusted *P* < 0.01. **a** Scatter plot of Log_2_ Fold Changes detected in response to London and Murcia spider mite strains. **b** Parametric analysis of gene set enrichment (PAGE) network based on Biological Processes (BP) Gene Ontology (GO) annotation with significantly enriched up- and down-regulated gene sets. Nodes represent gene sets, edges indicate the overlap in genes belonging to connected gene sets. Gene sets: blue – down-regulated, red – up-regulated, gray – not detected as differentially regulated. Size corresponds to number of genes in a given gene set (5 to 163), correspondence between node labels and GO Term/ID is provided in Data S6. The color (gray to red) and width of the edges correspond to an overlap size (3 to 43)

The overwhelming difference in number of DEGs induced by London and Murcia strains (390 and 4,205 DEGs respectively) may have obscured the identity of the grapevine responses to London mites in the GO and the GSA analyses, as they were based on DEGs induced by either of the two strains. To specifically check the nature of grapevine responses to non-adapted London mite strain, the cut-off of calling DEG was relaxed by applying only the BH corrected *p* value below 0.01, without applying the fold-change filter. This analysis detected 1116 DEGs as a grapevine response to herbivory of London mites, Additional file 5. Nevertheless, similar to the analysis of a smaller data set of 390 DEGs, the grapevine responses to London mite strain were substantially diminished relative to the induction of these genes by the Murcia mites, Fig. 4a. The PAGE network of the relaxed grapevine responses to London mite strain identified 89 biological processes (21 up- and 68 down-regulated), Fig. 4b (correspondence between node labels and GO Term/ID is provided in Additional file 6). Of 21 up-regulated processes, only 3 were significantly induced by London mites (nodulation (node 7), response to fungus (16) and response to wounding (18), Additional file 6), due to the modest change in levels of gene expression induced by London mites. In contrast, the great amplitude of expression of these genes in response to Murcia strain resulted in the significant up-regulation of all 21 processes. The identity of up-regulated gene sets points towards the establishment of a defense response to herbivore attack as they included responses to JA (node 15), ET (13), wounding (18) and chitin (14), as well as the biosynthesis of coumarin (6) and stilbene (5), Fig. 4b and Additional file 6. In the case of down-regulated gene sets, similar to results obtained at the stricter cut-off shown in Fig. 3, photosynthesis, plant growth and cell proliferation were general processes identified. Of these, the plant growth-associated biological processes (nodes 47–56) did not significantly change in response to London mites, while photosynthesis (22–46) and cell proliferation (57–89) did, albeit supported by the lower number of categories relative to the response suppressed by the Murcia strain. Thus, the ability of grapevine to successfully deter feeding of non-adapted London mite strain is associated with limited (in number of DEG) and weak (in amplitude of change) induced responses that show a signature of defense.

### Grapevine responses to feeding of adapted Murcia mite strain

**Fig. 5:**
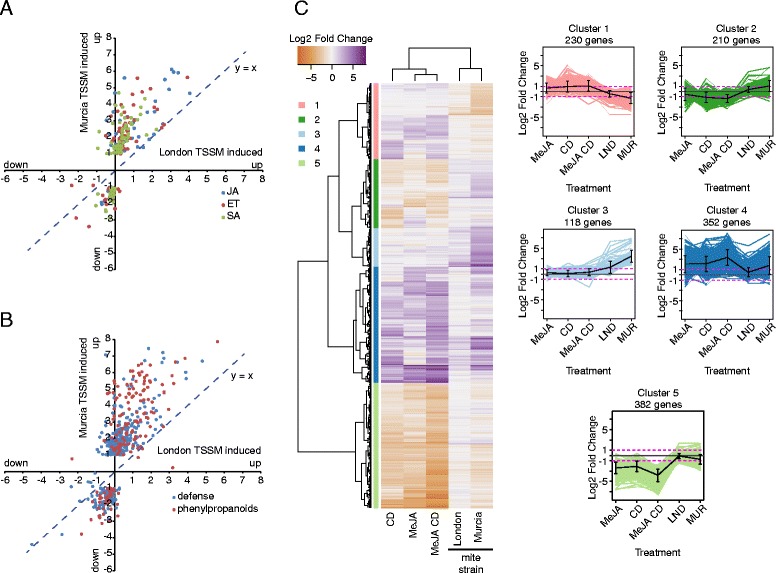
Analysis of differentially expressed genes associated with hormone signaling and defense compound production in response to spider mite feeding and comparison of grapevine response to JA pathway induction and spider mite feeding. **a** Scatter plot of Log_2_ Fold Changes of DEG associated with JA (blue), ET (red) and SA (green) signaling cascades detected in response to London and Murcia spider mite strains. **b** Scatter plot of Log_2_ Fold Changes of DEG associated with defense compounds (blue) and phenylpropanoids (red) production detected in response to London and Murcia spider mite strains. **c** Hierarchical clustering analysis of log_2_ Fold Changes of 1292 genes detected as most variable in response to induction of JA pathway [16] and spider mite feeding (this study). Genes and condition clustering was performed using Pearson’s *r* as a distance metric and average clustering

Murcia mites feed extensively, Fig. 1, inducing a robust grapevine response, Figs. 2, 3 and 4, including induction of genes encoding enzymes involved in the biosynthesis of JA, ET and SA, as well as many defense proteins (e.g. polyphenol oxidases, proteinase inhibitors, acid phosphatases, chitinases, peroxidases, numerous disease-resistance and pathogenesis-related proteins, and receptor-like proteins) and defense metabolites such as stilbenes, Fig. 5a, b and Additional file 7. These genes are characteristic for grapevine responses to biotic and abiotic stresses [16–21] and their homologues were previously identified in *Arabidopsis* and/or tomato defense responses against feeding of spider mites that were not adapted to these plants [4, 5]. Thus, Murcia mites induce grapevine responses that have a strong signature of defense.

Despite the induction of the robust grapevine defense responses, successful establishment of Murcia mite population on grapevine indicates that these responses are ineffective to substantially limit their fitness. To gain further insights into Murcia-induced responses, we compared them with grapevine responses to methyl-jasmonate (MeJA) and cyclodextrins (CD) [16]. Application of MeJA mimics naturally occurring increase in JA concentrations induced by TSSM herbivory [5] and CD triggers signal transduction cascade that results in the induction of genes involved in the stilbene synthesis and downregulation of programs associated with cell growth and division, also observed in response to TSSM feeding, Fig. 3. A total of 1,292 genes, clustered in 5 groups, demonstrated a high degree of variability across treatments, Fig. 5c and Additional file 8. Genes in cluster 1 were down-regulated only in response to mite treatments. These genes are associated with photosynthesis, a process that is altered in cultured cells used for the application of MeJA and CD, thus, the difference in the expression of these genes likely results from the nature of samples used. Clusters 2, 3 and 4 contain genes that are associated with various defense-related processes: genes in cluster 2 have a tendency toward down-regulation in response to MeJA and CD, but up-regulation in response to mites, while genes in clusters 3 and 4 are up-regulated by either spider mite feeding (cluster 3) or more prominently by MeJA and CD (cluster 4), indicating that while MeJA/CD and mites trigger similar differential expression of many common genes, there are also gene sets that are differentially regulated by these treatments. Finally, cell division and growth are commonly down-regulated across treatments, but MeJA and CD seem to affect greater number of these genes that are grouped in cluster 5. Thus, meta-analysis identified differences in the gene expression triggered by MeJA/CD and mite herbivory. However, none of the clusters displayed the expression pattern expected for the suppression of grapevine defenses by adapted mites. Responses induced by the London- and the Murcia-mites had similar trends and clustered together, indicating that differences between treatments resulted either from differences in samples used or were reflection of differential contribution of MeJA/CD and mite herbivory to the commonly affected processes.

### Core plant responses induced by spider mites

**Fig. 6:**
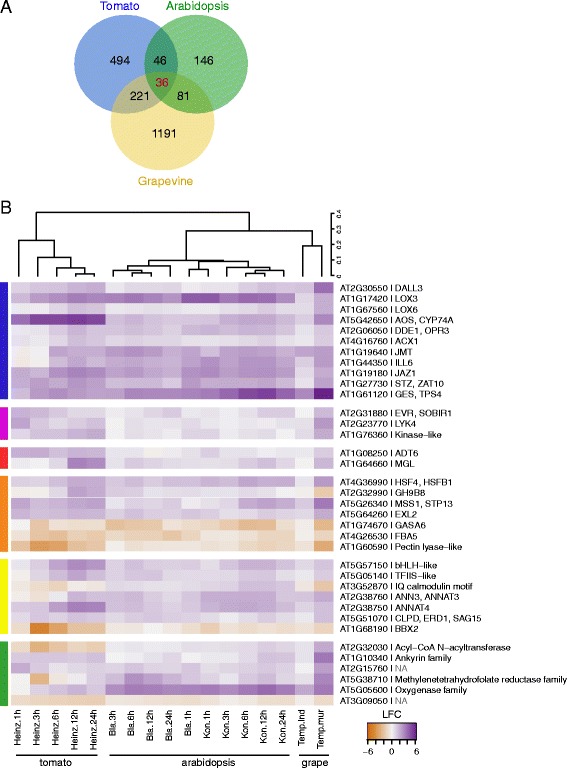
Analysis of conservation of defense response to spider mite herbivory across *Arabidopsis*, grapevine and tomato. **a** Comparison of DEG detected in response to spider mite herbivory that are orthologous across three species [4, 5]. **b** Hierarchical clustering analysis of log_2_ Fold Changes of core 36 conserved DEG involved in JA metabolism and signaling (blue), perception (purple), amino acid metabolism (red), growth (orange), regulation (yellow) and enzymatic reactions (green)

We have previously identified 1,109 *Arabidopsis* and 2,133 tomato genes as differentially expressed in response to spider mite herbivory [4, 5]. Complementing these data with 4,205 grapevine DEGs identified in this study, allows identification of the conserved responses to spider mite feeding across three phylogenetically diverse plant species. We have first identified a total of 9,305 trios of putative bidirectional best hit (BBH) orthologous between *Arabidopsis*, tomato and grapevine (Additional file 9). Of these, only 309, 797 and 1529 were differentially expressed in *Arabidopsis*, tomato and grapevine respectively upon mite herbivory, indicating that the majority of DEGs did not have orthologous genes across species examined. Of the orthologous genes, a fraction was identified as DEG in response to mite herbivory in more than one species (52 % in *Arabidopsis*, 38 % in tomato and 22 % in grapevine), with a limited core of 36 orthologous genes that were differentially expressed and in the same direction across all three species, Fig. 6a. Consistent with the established conserved role of JA in regulation of plant responses to herbivory, the core group includes genes involved in JA biosynthesis (*DAD1-LIKE LIPASE 3 (DALL3), LIPOXYGENASE (LOX3* and *6), ALLENE OXIDE SYNTHASE (AOS), OXOPHYTODIENOATE-REDUCTASE 3 (OPR3), ACYL-COA OXIDASE 1 (ACX1)),* metabolism (*JASMONIC ACID CARBOXYL METHYLTRANSFERASE (JMT) and IAA-LEUCINE RESISTANT (ILR)-LIKE GENE 6 (ILL6)),* regulation and signaling (*JASMONATE-ZIM-DOMAIN PROTEIN 1 (JAZ1), SALT TOLERANCE ZINC FINGER (ZAT10*)) and response (*TERPENE SYNTHASE 4 (TPS04))* [28–31]*,* Fig. 6b. In addition, supporting the potential importance of chitin(−like) elicitors of mite feeding and involvement of LRR-receptor-like proteins (RLP), two receptor kinases, *LYSM-CONTAINING RECEPTOR-LIKE KINASE 4 (LYK4*) and *SUPPRESSOR OF BIR1-1 (SOBIR1*) were up-regulated across plant species, as well as the kinase encoded by ortholog of uncharacterized *AT1G76360* locus that was recently linked to plant responses to UV radiation [32–35]. Further, core includes orthologous genes encoding *METHIONINE GAMMA-LYASE* (*MGL*), involved in cellular methionine homeostasis and biosynthesis of isoleucine (Ile), and *AROGENATE DEHYDRATASE* (*ADT6*) that catalyzes the final step in phenylalanine (Phe) biosynthesis [36, 37]. Isoleucine is required as conjugant for the biosynthesis of biologically active JA-Ile metabolite, while phenylalanine is a precursor for the biosynthesis of the defensive phenylpropanoid class of metabolites [38], indicating the shift from the primary to the secondary metabolisms as a response to herbivory. Core genes also reflect the conservation of the transition from plant growth to plant defense that is associated with plant responses to biotic stress [39]. In *Arabidopsis*, this transition is orchestrated by the *HEAT SHOCK FACTOR 4 (HSF4)*, a transcription factor whose expression was up-regulated by mite feeding across all three species that acts to repress genes encoding chloroplast proteins [40]. Arrest of the plant growth is also associated with changes in the sugar metabolism (illustrated by the up-regulation of a *SUGAR TRANSPORT PROTEIN (STP13)* required for the retrieval of hexoses from the apoplast across the plasma membrane [32] and cytosolic *FRUCTOSE 1,6-BISPHOSPHATE ALDOLASE (FBA5)* [41] involved in the glycolysis), changes in the cell wall properties (seen through the induction of *CELLULASE (GH9B8),* down-regulation of *PECTIN LYASE* and expression of genes such as *EXORDIUM-LIKE (EXL2*), a repressor of growth that is responsive to diminishing energy status in the cell, and *GA-STIMULATED ARABIDOPSIS 6 (GASA6),* a hypothetical secreted peptide hormone precursor associated with cell growth [42, 43]). Core also includes regulatory proteins ranging from transcriptional regulators (*AT5G57150, AT5G05140* and *B-BOX DOMAIN PROTEIN 27 (BBX27)* that is down-regulated by spider mite herbivory), proteins mediating Ca^2+^ signaling (*ANNEXINS* 3 and 4 (*ANN3* and *4*) and AT3G52870), and *EARLY RESPONSIVE TO DEHYDRATION 1 (ERD1),* a Hsp100 chaperone involved in protein quality control and protein import in chloroplasts. Finally, there is a class of proteins that encode enzymes, none of which are currently associated with specific substrates. Thus, the conserved core identifies genes involved in known processes, but also genes whose function is still not understood.

## Discussion

Grapevine shows a host-resistance toward the non-adapted London strain. Efficient grapevine defenses are reflected in mite dispersal and limited feeding that is associated with the induction of weak host transcriptional responses. On the opposite end of the adaptation spectrum, Murcia mites are retained on grapevine leaves, they feed intensively and trigger prominent plant responses. Grapevine responses induced by these strains are similar in identity, but differ in levels that are proportional to damage inflicted, Figs. 1, 2, 3 and 4. Induced grapevine responses are characterized by the down-regulation of photosynthesis, cell division and growth, and an up-regulation of genes involved in biosynthesis, signaling and responses to JA, ET and SA, that were also observed in *Arabidopsis* and tomato responses to mite feeding [4, 5, 12]. Despite the conservation of these programs across three species, the majority of DEGs and defense compounds synthesized are species specific and reflect differences in secondary metabolism between these plants. While *Arabidopsis* defenses against spider mite herbivory rely on indole glucosinolates [5], tomato defenses are mostly based on the anti-digestive proteins such as *proteinase inhibitors* (*PI*), *leucine amino peptidase (LAP*), *threonine deaminase (TD*), and *polyphenol oxidases* (*PPO*) [4]. The grapevine transcriptional responses to mite herbivory capture both anti-digestive proteins and defensive metabolites. For example, induced expression of *PIs* and *PPOs* in response to mite feeding is common between tomato and grapevine, but not in *Arabidopsis* where they are either lacking in the genome (*PPO*s) [44] or were not recruited for defense (*PIs*) [4]. PIs were shown to be effective in restricting mite herbivory [45, 46], however, PPO*s* are likely ineffective in the mite’s acidic gut [47]. In addition, the major metabolic output of grapevine-induced defenses is the production of stilbenes, phenylpropanoid metabolites derived from phenylalanine [16]. Even though genes encoding phenylpropanoid biosynthetic enzymes are induced upon mite herbivory in all three species, *stilbene synthase (STS)* that allows the synthesis of stilbenes is only present in the grapevine genome, being absent in both tomato and *Arabidopsis* [48]. Antifungal and antimicrobial activities of stilbenes are well characterized [49–51], however, their role in defense against herbivores is not clear. A spruce bark beetle, *Ips typographus,* associates with fungus *Ceratocystis polonica* in order to feed on Norway spruce, a plant species that also accumulates defensive stilbenes. The fungus is capable of degrading stilbenes [52], making it plausible that its detoxification may also benefit bark beetles. Stilbenes may be part of the functional output of grapevine defenses against spider mites, a hypothesis that should be tested by using the *Arabidopsis* or tomato transgenic plants that constitutively express heterologous *STS* genes and synthesize stilbenes [53, 54].

The prominence of grapevine induced transcriptional responses correlated with the feeding intensity, Figs. 1 and 2. This is an opposite pattern to one observed for tomato responses to feeding of tomato-adapted and non-adapted TSSMs [12]. In this particular case, the non-adapted mites triggered robust responses expected to be efficient in restricting mite feeding, and tomato-adapted mites induced similar but attenuated transcriptional changes. Suppression of plant defenses by herbivores has been described in several cases and is mediated through secretion of salivary effectors at the feeding site where they interfere with plant responses [14, 55–60]. Reprograming of grapevine development, defenses and metabolism has been documented in the case of phylloxera *(Daktulosphaira vitifoliae),* a grapevine specialist and leaf-galling herbivore that induces ectopic formation of stomata and alters source-sink metabolism at the feeding site [22]. We examined a possibility that Murcia-adapted mites manipulate grapevine responses by performing a meta-analysis that compared responses triggered by MeJA/CD and mite herbivory, but did not identify expression patterns expected for the suppression of grapevine responses by adapted mites, Fig. 5. In the absence of characterized effective grapevine defenses against spider mites, the functionality of Murcia-induced responses remains elusive. If Murcia-induced responses are effective to restrict herbivory of non-adapted mites, as suggested by its similarity to the effective *Arabidopsis* and tomato defenses, then Murcia strain evolved the ability to overcome them. It has been shown that mites can evolve resistance to pesticides or new hosts through reprograming of their xenobiotic metabolism within 10–30 generations [12, 61, 62], making it plausible that detoxification of grapevine defense compounds underlies adaptation of Murcia mites to grapevine. In this case, it is unclear if dampened grapevine responses triggered by London strain (Figs. 2, 3 and 4) are able to restrict its herbivory, or grapevine-resistance to London mites relies on potent constitutive defenses. The nature and the identity of potential constitutive defenses in grapevine are unknown, however, cuticle, trichomes and metabolites such as acylsugars, methyl ketones, terpenoids and cyanogenic glucosides cause mite mortality or deter mites from feeding on gorse, wild tomato relatives and beans [8, 13, 63–70].

Comparison of spider mite-induced responses between grapevine, tomato and *Arabidopsis* identified a core of 36 one-to-one orthologous genes, Fig. 6. Consistent with the conserved role of JA in regulating defenses against mite herbivory [4, 5], JA biosynthetic and signaling genes are included in this data set. In addition, orthologs of *SOBIR1* and *LYK4* receptors, previously identified in *Arabidopsis* and tomato responses to mite feeding [4] are also present in the grapevine data set. In *Arabidopsis*, *LYK4* facilitates the recognition of chitin-related ligands by *LYK5* [33]. *LYK5* is induced by mite feeding in *Arabidopsis*, however, its tomato and grapevine orthologs were not [4, 5]. In rice, chitin perception is mediated by the chitin-elicitor binding protein (CEBiP) [71–73], which contains an extracellular LysM motif and a transmembrane domain, but lacks an intracellular kinase domain. Thus, there is a possibility that some other plasma membrane-associated LysM motif-containing proteins contribute to chitin perception in tomato and grapevine. Chitin is the main component of the exoskeleton and gut lining in arthropods [74]. A conceivable elicitation of plant responses by mite-originating chitin is potentiated by the induction of chitinases upon mite feeding in all three plant species. Chitinases are glycosyl hydrolases required for the chitin breakdown and the production of chitin oligomers that act as ligands. Chitinases are used by arthropod pathogens to hydrolyze exoskeletal chitin, to aid in penetration of their hosts (e.g. *Beauveria bassiana* that is also a pathogen of *T. urticae* [75, 76]). In addition, chitinases administered through an artificial diet that target chitin in the arthropode gut, were shown to affect growth and development of a wide range of pests [77, 78]. Significantly, plant chitinases require an acidic environment for their activity [79]. In contrast to the alkaline pH of Lepidopteran guts, the mite gut is acidic [80]. Therefore, plants may be able to generate and recognize mite-associated chitin oligomers, a possibility that should be challenged in the future.

## Conclusions

This study describes genome-wide grapevine transcriptional changes triggered by the grapevine-adapted and the non-adapted spider mite strains. The adapted mites induced robust plant responses that captured biological processes previously associated with effective defenses against mite feeding in *Arabidopsis* and tomato. However, it remained elusive if pronounced grapevine transcriptional reprograming in response to the adaptive strain merely associates with the greater tissue damage or it establishes defense that adapted mites can detoxify. In contrast, the non-adapted mites induced similar grapevine responses but of lower magnitude that clustered with untreated control. Association of host-resistance that efficiently restricts the performance of non-adapted mites with responses that are limited both in numbers of DEGs and the amplitude of their induction, raises a possibility that constitutive responses may present a barrier to feeding of the non-adapted spider mites. Resolution of the effectiveness of grapevine responses to mite feeding and the corresponding adaptation mechanism(s) evolved by Murcia mites to override feeding restrictions necessitates the identification of additional independent patterns of grapevine responses to both adapted and non-adapted mite strains in future.

Comparison of the spider mite responses induced in grapevine, *Arabidopsis* and tomato identified the conserved core of 36 orthologous genes that were differentially expressed in all three species. Consistent with the established conserved role of JA in regulation of plant responses to herbivory, the core group includes genes involved in JA biosynthesis and signaling. In addition, core also included orthologous receptors associated with chitin perception, raising a possibility that plants recognize mite-associated chitin oligomers.

## Methods

### *Tetranychus urticae* strain selection

Murcia strain was collected from heavily infested vineyard of *Vitis vinifera* L. cv. ‘Crimson Seedless’ in Alhama de Murcia (Región de Murcia, Spain). The taxonomic status of the Murcia TSSM strain has been confirmed through the shape of male aedeagus [26]. Inbred lines were developed for both London and Murcia strains from isofemale lines that underwent eight and seven consecutive generations of mother-son matings respectively. Murcia mites were mass reared in the laboratory on potted *Vitis vinifera* L. cv. ‘Tempranillo’, while London mites were reared on bean plants (*Phaseolus vulgaris* ‘California Red Kidney’; Stokes) in growth chambers at 25 °C ± 1 °C with a 16:8 h (light/dark) photoperiod. To eliminate the effect of rearing plant hosts on physiological state of mites, both Murcia and London mites were kept for two generations on bean leaves prior to transferring them to the experimental grapevine plants.

### Preparation of grapevine plantlets

A protocol for production of healthy and physiologically uniform grapevine plantlets has been developed and is described in Additional file 1 [81, 82]. Dormant grapevine cuttings (cv. Tempranillo) have been collected from the field-grown vines in Logrono, La Rioja, in December of 2013 and were kept at 4 °C until the propagation.

### Damage assay

A single leaf on a plant with 1–2 leaves was isolated with wet cotton and lanolin barrier 24 h ahead of the experiment and was inoculated with 50 female mites of either Murcia (reared for two generations on bean) or London TSSM strain. Untreated plants were used as a control. After feeding for 24 h, leaves were cut and scanned with transmitted light using an Epson Scan V370 Photo with film adapter (Epson, Suwa, Japan) using following settings: document type—film; type of film—positive color; resolution—1200 dpi. Leaf images were processed using the Gimp software v2.6 (http://www.gimp.org/) that allowed selection and quantification of all the brownish spots that were associated with mite feeding. Signal that was considered as damage was negligible on untreated leaves. Leaf damage data was analyzed using factorial ANOVA to assess significance of the mite strain and experimental block effects. ANOVA was followed by Tukey’s Honestly Significant Difference test.

### Dispersal assay

A single leaf on a plant with 1–2 leaves was inoculated with 50 female mites of either Murcia (reared for two generations on bean) or London TSSM strain. Number of mites retained on the inoculated leaf after 24 h was determined. Proportion of mites retained was arcsine transformed and factorial ANOVA was used to assess significance of the mite strain and experimental block effects. ANOVA was followed by Tukey’s Honestly Significant Difference test.

### Preparation of samples for transcriptome analysis

Plants with developed first two leaves were grown under 100 to 150 μmol m^−2^ s^−1^ cool-white fluorescent light at 26 °C with a 16 h/8 h (light/dark) photoperiod in controlled growth chambers. Petioles of experimental leaves were surrounded with wet cotton and lanolin to confine mites on the inoculated leaves. The barrier was placed 24 h ahead of mite inoculation and on all experimental plants in order to unify potential effects of this plant manipulation. 50 adult female spider mites of either Murcia or London strain were applied per plant and allowed to feed for 24 h. Untreated plants were used as control. Four biological replicates containing leaves from two plants were generated per treatment. Treated leaves were collected, frozen in liquid nitrogen and kept at −80 °C until they were used for RNA extraction. Total RNA was extracted from frozen tissues using the Spectrum Plant Total RNA Kit (Sigma-Aldrich) and performing on-column DNase I digestion according to manufacturer’s protocol to avoid DNA contamination.

### RNA-Seq analysis of grapevine responses to spider mite feeding

RNA samples were processed to construct strand-specific cDNA libraries (one per biological sample) using Illumina TruSeq RNA Library Preparation Kit (Illumina, San Diego, CA). Sequencing of all 12 libraries was conducted on a single sequencing lane using Illumina HiSeq 2000 platform (Illumina, San Diego, CA) to produce 4.2–5.2 million strand-specific 100 bp paired-end reads per library. Reads were mapped to the reference (12X) grapevine genome using STAR aligner [83] allowing only for unique mapping and up to two mismatches per read mapped, using v.2.1 gene prediction provided by CRIBI Biotechnology Center, University of Padua. Read counts were generated using HTSeq at the level of gene locus [84]. Analysis of differential gene expression was performed using voom/limma workflow for genes that demonstrated expression level of at least 1 count per million (CPM) in at least 4 samples [85].

### Gene set analysis

Gene set analysis (GSA) was performed using a custom version of Bioconductor package piano [86]. Log_2_ FC, *p*- and *t*-values obtained using limma/voom were used as the input gene level statistics for analysis utilizing the PAGE algorithm [87]. Biological Process Gene Ontology annotation was used to classify genes into sets. We used a combination of automated Blast2GO annotation provided by CRIBI Biotechnology Center, University of Padua and V1 curated annotation [88] to identify biological processes enriched in DEGs, see Additional file 10 for final GO annotation file.

### Gene ontology analysis

GO analysis was performed using topGO with Fisher’s test statistic and “weight01” algorithm [89] to generate a list of top 50 Biological Process GO annotations and annotate lists of genes that were detected as differentially expressed. The lists were further filtered by applying a cut-off of 0.05 to Fisher’s weighted *p*-values.

### Comparison of grapevine responses to spider mite feeding and those induced by the application of JA and/or elicitors

CEL files for grapevine induced JA response [16] were retrieved from PLEXdb (accession vv44) [90]. Expression measures were computed using RMA [91] and Log_2_ fold changes for all genes and for all relevant contrasts were calculated using limma [92]. Correspondence between Affymetrix probe ID and current grapevine genome annotation was established according to Grimplet et al., 2012 [88]. To retain genes that were informative for the inference of relatedness between effects of JA and elicitors and spider mite herbivory we have selected a sample standard deviation cut-off of Log_2_ fold change across compared experiments and contrasts equal to 1. We retained a data matrix of 1,292 genes (Additional file 8) and their respective Log_2_ fold changes across experiments for subsequent study comparison.

### Establishment of bidirectional best hit orthologs between grapevine, tomato and *Arabidopsis*

To determine one-to-one orthologs using the bidirectional best hit (BBH) approach [93], reciprocal blastp [94] searches were conducted using v.2.1 release of grapevine provided by CRIBI Biotechnology Center, ITAG v.2.3 release of tomato and TAIR10 release of *Arabidopsis* protein sequences with cut-off of E < 10^−4^. Output files were further processed to retain BBH trios, listed in Additional file 9.

## Availability of supporting data

The transcriptome data set supporting the results of this article is available through the Sequence Read Archive (SRA) accession SRP067967. In addition, the data sets supporting the results of this article, as well as Additional Method, are included as additional files.

## Additional files

Additional file 1:
**Method-Preparation of grapevine plantlets.** (DOCX 811 kb) Additional file 2:
**DEGs detected with absolute FC > 2 and adjusted** ***p*** **< 0.01 in response to feeding of grapevine-adapted and non-adapted mite strains.** (XLSX 1803 kb) Additional file 3:
**GSA results for the DEGs detected with absolute FC > 2 and adjusted** ***p*** **< 0.01 in response to spider mite herbivory in at least one of the treatments.** (XLSX 128 kb) Additional file 4:
**DEGs detected with absolute FC > 2 and adjusted** ***p*** **< 0.01 only in the response to the feeding of London mites.** (XLSX 30 kb) Additional file 5:
**DEGs detected with an adjusted** ***p*** **< 0.01 in response to the feeding of London mites.** (XLSX 353 kb) Additional file 6:
**GSA results for the DEGs detected with an adjusted** ***p*** **< 0.01 in response to the feeding of London mites.** (XLSX 36 kb) Additional file 7:
**List of DEGs and their FC values associated with the hormone and phenylpropanoid metabolism and defense.** (XLSX 150 kb) Additional file 8:
**List of DEGs and their FC values used for the comparison of grapevine responses to MeJA, CD and spider mite feeding.** (XLSX 336 kb) Additional file 9:
**List of** ***Arabidopsis*** **-tomato-grapevine orthologous trios.** (XLSX 749 kb) Additional file 10:
***Vitis vinifera*** **locus annotation used in this study.** (XLSX 3659 kb)
