# Supplementary material for: Comparative genome-wide transcriptome analysis of Vitis vinifera responses to adapted and non-adapted strains of two-spotted spider mite, Tetranyhus urticae
Source: BMC Genomics. 2016 Jan 22;17:74. doi: 10.1186/s12864-016-2401-3 (PMC4724079; doi:10.1186/s12864-016-2401-3)
Supplement: Additional file 1: — Method-Preparation of grapevine plantlets. (DOCX 811 kb) [file 12864_2016_2401_MOESM1_ESM.docx]

**Methods S1: Preparation of grapevine plantlets**

Preparation of grapevine plantlets proceeds in several stages as shown in Figure 1. Initially, grapevine cuttings are cleaned from the potential contaminants from field (mainly fungi), then cuttings are rooted to provide good support for the development of the shoot bud, and finally the bud development is induced.


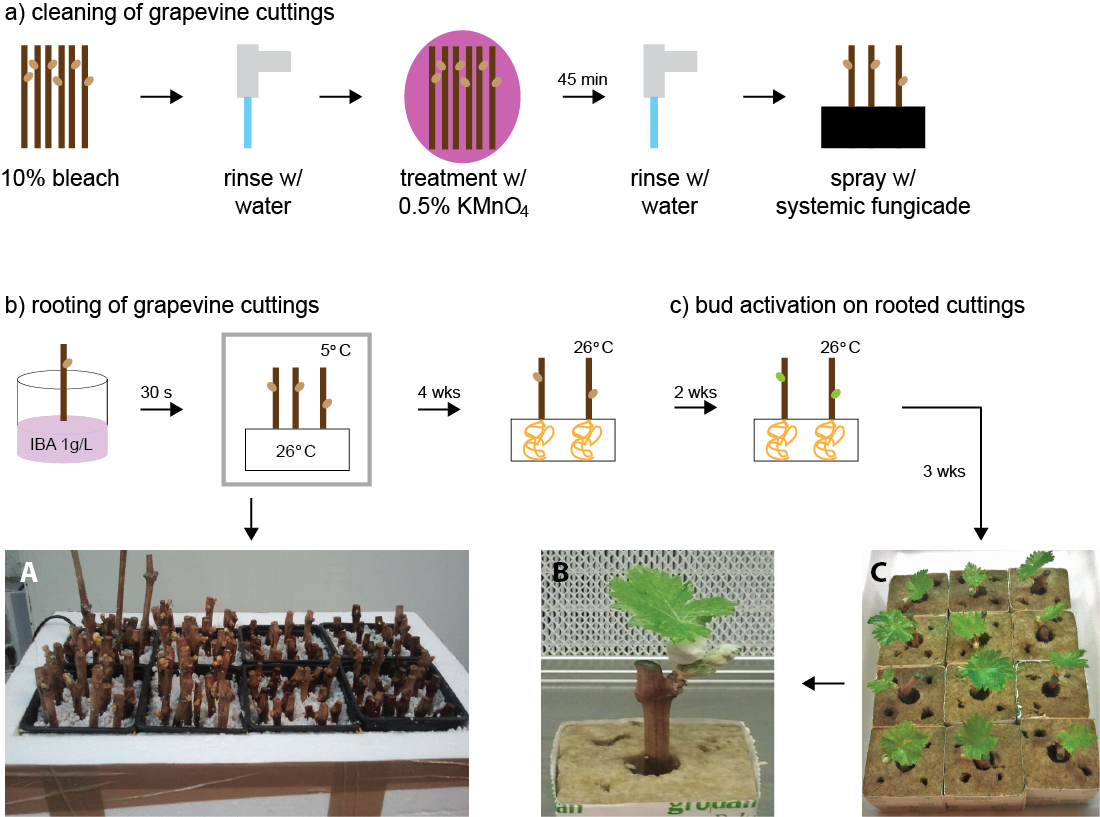


**Figure 1.** Preparation of plantlets from field collected cuttings of grapevine. Schematics show three stages in plantlet propagation: a) cleaning of the grapevine cuttings; b) rooting of the grapevine cuttings; and c) bud activation of rooted cuttings. A, Experimental set-up for rooting of grapevine cuttings. B and C, produced plantlets.

**a) Cleaning grapevine cuttings**

Dormant grapevine cuttings, collected from the field-grown vines were stored at 4 ^o^C until the propagation. Cuttings, 10 cm long containing a single bud, were washed thoroughly with a metallic sponge in 10% bleach (v/v) and were rinsed with tap water. Subsequently, cuttings were surface sterilized by submerging them in 0.5% KMnO_4_ (PanReac AppliChem, ES) for 45 minutes. Upon washing, cuttings were sprayed with systemic fungicide (1% Benomyl, methyl N-[1-(butylcarbamoyl)benzimidazol-2-yl] carbamate, [Sigma-Aldrich](http://www.sigmaaldrich.com?cm_mmc=affiliate-_-PubChem-_-product-_-link)) and were left overnight. These treatments were all performed at room temperature and can be completed in one day.

**b) Rooting of grapevine cuttings**

To promote rooting, the bottom part of the cleaned grapevine cuttings were dipped in the solution of indolebutyric acid (IBA) at the concentration of 1g/l for 30 secs, before they were fitted in rockwool cubes (Grodan delta cube 7.5cm, GRODAN Group, The Netherlands). The bottom portion of the cutting was kept at 26 ^o^C, while the top was maintained at 5 ^o^C in dark, in order to promote rooting but to retain bud dormancy ([Alley, 1979](#_ENREF_1)). This is achieved by keeping cuttings for four weeks in cold room (4-5^o^C) in Styrofoam box fitted with the heater at the bottom, see Figure 1A.

**c) Activation of bud growth on rooted grapevine cuttings**

After 4 weeks, rooted cuttings were transferred to the growth chamber (25 °C, 16 h/8 h (light/dark) photoperiod). Two weeks later, budbreak took place and cuttings were grown for 2-3 more weeks to reach the stage used for experiments (5cm long sprouts with 1-2 leaves of 3-5cm diameter), see Figures 1B and C. During growing, rockwool cubes were kept wet and supplemented with modified Hoagland solution ([Wheatley *et al.*, 2009](#_ENREF_2)).

**References**

**Alley, C.J.** (1979) Grapevine Propagation .11. Rooting of Cuttings - Effects of Indolebutyric Acid (Iba) and Refrigeration on Rooting. *Am J Enol Viticult*, **30**, 28-32.

**Wheatley, M.D., Tattersall, E.A.R., Tillett, R.L. and Cramer, G.R.** (2009) An Expanded Clay Pebble, Continuous Recirculating Drip System for Viable Long-Term Hydroponic Grapevine Culture. *Am J Enol Viticult*, **60**, 542-549.
